# Nanobody-based chimeric antigen receptor T cells designed by CRISPR/Cas9 technology for solid tumor immunotherapy

**DOI:** 10.1038/s41392-021-00462-1

**Published:** 2021-02-25

**Authors:** Fengzhen Mo, Siliang Duan, Xiaobing Jiang, Xiaomei Yang, Xiaoqiong Hou, Wei Shi, Cueva Jumbo Juan Carlos, Aiqun Liu, Shihua Yin, Wu Wang, Hua Yao, Zihang Yu, Zhuoran Tang, Shenxia Xie, Ziqiang Ding, Xinyue Zhao, Bruce D. Hammock, Xiaoling Lu

**Affiliations:** 1 International Nanobody Research Center of Guangxi, Guangxi Medical University, Nanning, Guangxi 530021 China; 2 Pharmaceutical College, Guangxi Medical University, Nanning, Guangxi 530021 China; 3 Department of Neurosurgery, Union Hospital, Tongji Medical College, Huazhong University of Science and Technology, Wuhan, Hubei 430022 China; 4 School of Preclinical Medicine, Guangxi Medical University, Nanning, Guangxi 530021 China; 5 College of Stomatology, Guangxi Medical University, Nanning, Guangxi 530021 China; 6 Department of Entomology and Nematology and UCD Comprehensive Cancer Center, University of California Davis, Davis, CA 95616-8584 USA

**Keywords:** Tumour immunology, Tumour immunology

## Abstract

Chimeric antigen receptor-based T-cell immunotherapy is a promising strategy for treatment of hematological malignant tumors; however, its efficacy towards solid cancer remains challenging. We therefore focused on developing nanobody-based CAR-T cells that treat the solid tumor. CD105 expression is upregulated on neoangiogenic endothelial and cancer cells. CD105 has been developed as a drug target. Here we show the generation of a CD105-specific nanobody, an anti-human CD105 CAR-T cells, by inserting the sequences for anti-CD105 nanobody-linked standard cassette genes into AAVS1 site using CRISPR/Cas9 technology. Co-culture with CD105^+^ target cells led to the activation of anti-CD105 CAR-T cells that displayed the typically activated cytotoxic T-cell characters, ability to proliferate, the production of pro-inflammatory cytokines, and the specific killing efficacy against CD105^+^ target cells in vitro. The in vivo treatment with anti-CD105 CAR-T cells significantly inhibited the growth of implanted CD105^+^ tumors, reduced tumor weight, and prolonged the survival time of tumor-bearing NOD/SCID mice. Nanobody-based CAR-T cells can therefore function as an antitumor agent in human tumor xenograft models. Our findings determined that the strategy of nanobody-based CAR-T cells engineered by CRISPR/Cas9 system has a certain potential to treat solid tumor through targeting CD105 antigen.

## Introduction

It is known that malignant tumors are able to invade and metastasize due to their ability to escape the immune system surveillance. CD105, also termed endoglin, is highly expressed on cancer cells, and on peri- and intratumoral endothelial cells, which line tumor blood vessels.^1,2^ On the other hand, CD105 is expressed in varying degrees in the vasculature of normal tissues and on normal blood vessels, except for the umbilical cord of newborns.^3^ A phase II study of TRC105 in patients with hepatocellular carcinoma (HCC) or advanced/metastatic urothelial carcinoma were carried out by Tracon Pharma and several oncologists. Although TRC105 was well tolerated, it did not improve progression-free survival in patients, and thus suggest that TRC105 therapy will not be effective as a single agent using the present schedule of tumor treatment. Common adverse effects included infusion reactions, headache, epistaxis, and oral hemorrhage during TRC105 treatment of solid tumors.^4,5^ At present, multiple clinical trials to evaluate the potential of TRC105 in combination with vascular endothelial growth factor or programmed cell death protein 1 (PD-1) checkpoint inhibitors are being carried out and some have obtained positive effects.^6–8^ Compelling evidence support the notion that CD105 plays a key role in angiogenesis, and that CD105 is an optimal molecular target.^9,10^ Therefore, CD105 can be used to design innovative biological immunotherapy to treat human malignant tumors.

Adoptive immunotherapies based on chimeric antigen receptor T (CAR-T) cells provide promising therapeutic approaches for the treatment of tumor diseases, in particular hematological malignancies.^11,12^ Commonly, CAR-T cells contain a chimeric antigen that consists of a tumor antigen-specific single-chain variable fragment (scFv), hinge region, transmembrane region, and intracellular signal domains. CAR-T cells overcome the restriction of the human leukocyte antigen system to activate antitumor T cells.^13,14^ However, CAR-T cell-based immunotherapies for treating solid tumors remain challenging, mainly due to sub-optimal antigen recognition that is specific to tumor cells, inefficient trafficking of CAR-T cells to tumor sites,^15,16^ and the immunosuppressive effects of the tumor microenvironment.^17^ The therapeutic efficacy of CAR-T cell-based immunotherapies is limited by factors such as the potential off-target effects,^18^ cross-reactivity of single-chain antibodies^19^, and random gene insertion-related mutations^20^ in solid tumors. Therefore, CAR-T cell engineering for solid tumors need further development, to provide higher safety, affinity, and efficacy, as well as lower immunogenicity.

The antigen recognition region of CAR-T cells is usually a scFv, which is composed of heavy-chain variable fragments connecting to light-chain variable fragments through a flexible linker.^14^ However, scFvs do not always fold efficiently and tends to aggregation.^21^ Nanobodies (Nbs), also known as variable regions of heavy-chain antibodies (VHHs),^22^ are significantly smaller in size compared to traditional monoclonal antibodies (width 2.5 nm, length 4.2 nm)^23^ and have stable physiochemical properties, which allow them to tolerate extreme conditions, such as high pressure or acidity, while maintaining their high affinity to antigens.^24,25^ These characteristics allow Nbs to spread rapidly throughout the body after administration, achieving an excellent tissue distribution.^26^ Moreover, high sequence homology with human VH3 gene family provides the relatively lower immunogenicity of Nbs in humans.^27,28^ Using Nbs to form a CAR would overcome the shortages of CAR based on scFv from traditional antibodies, such as the complex folding and assembly steps, as well as the lower protein stability that might impair the function.^29^ Thus, Nbs could serve as an ideal antigen recognition domain for generating tumor antigen-specific CAR-T cells.^29,30^

Clustered regulatory interspersed short palindromic repeat (CRISPR) is a special family of DNA repeats that are widely distributed in the bacterial and archaeal genomes.^31^ As a novel and effective gene-editing technology, CRISPR/Cas9 system allows directly targeting a specific gene in the genome, leading to precise insertion, deletion, and gene replacement.^32,33^ This technique has been widely used for targeting the T-cell receptor α constant (TRAC) locus, to prevent endogenous T cell receptor (TCR) expression, to integrate the CAR gene within the TRAC locus^34^, or to edit the TCR gene for redirecting T cells towards a cancer antigen.^35^ The adeno-associated virus integration site 1 (AAVS1) is a locus within the *PPP1R12C* gene and has been considered as a safe harbor for robust expression of CAG promoter-driven transgenes, but has not been applied for CAR molecule construction.^36^ Accordingly, we designed to construct the integration of the CD105-specific CAR genes into the AAVS1 locus using the CRISPR/Cas9 system may generate anti-CD105 CAR-T cells that have high levels of stable expression of anti-CD105 Nb and potent cytotoxicity against CD105^+^ tumors.

This study aimed to screen an anti-CD105 Nb that recognizes CD105^+^ target cells and generate a Nb-based CAR-T cells specific for CD105 (anti-CD105 CAR-T cells) engineered by the CRISPR/Cas9 technology. Tumor cell killing efficacy of the CAR-T cells was then tested in vitro and in vivo. This study may provide a new strategy for the generation of Nbs and the development of Nb-based CAR-T cells engineered by CRISPR/Cas9 system, to target CD105 antigen in the tumor microenvironment.

## Results

### Library construction, expression, and purification of CD105 Nb

**Fig. 1:**
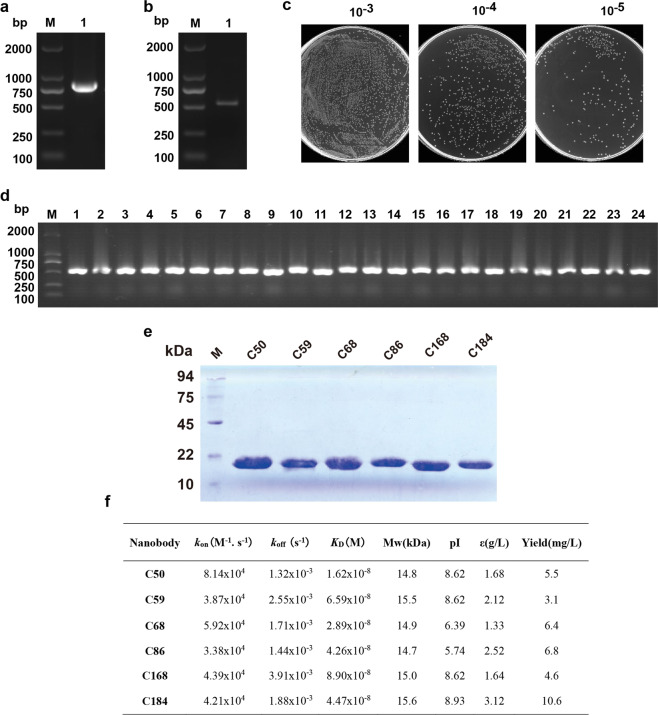
Library construction, expression, and purification of CD105 nanobody. **a**, **b** The VHH genes were obtained by two steps PCR. **c** The library size was measured by counting the colonies number after serial dilution. **d** Twenty-four colonies were randomly picked to estimate the correct insertion rate of VHH genes by PCR amplification. **e** SDS-PAGE analysis of purified CD105-specific nanobodies. **f** Properties of CD105-specific nanobodies. The affinity of these nanobodies was determined by Biacore. Theoretical isoelectric point (pI), molecular weight (Mw), and extinction coefficient (ε) were calculated by the ExPAsy ProtParam Tool

To construct the library, peripheral blood mononuclear cells (PBMCs) were isolated from the immunized camel and their total RNAs were extracted. The 700 bp fragments for the VH-CH2 regions were reversely transcribed into cDNA (Fig. 1a). The fragment was purified from gels as the template for PCR that generated products of 400 bp fragments for the VHH region (Fig. 1b). After being digested with PstI and NotI, the DNA fragments were cloned into the phagemid pMECS allowing the expression of C-terminal hemagglutinin-His6-tagged Nbs. Subsequently, the recombinant plasmid was transformed into competent TG1 cells by electroploration. The titer of this Nb library against CD105 was calculated by counting the number of colonies in gradient dilution plates (Fig. 1c), which showed that the library should have a probability to obtain Nbs with high specificity and sequence diversity. A total of 24 colonies was randomly chosen for PCR analysis and all libraries contained the desired insertion as shown in Fig. 1d. Subsequently, the recombinant plasmids were electro-transformed into WK6 cells to express the Nbs. SDS-polyacrylamide gel electrophoresis (SDS-PAGE) analysis displayed a band with approximately 15 kDa in molecular weight (Fig. 1e). These six Nbs had their equilibrium dissociation constants (*K*_D_) ranging from (1.62 ± 0.18) × 10^−8^ to (8.90 ± 0.27) × 10^−8^ M; the high-affinity Nbs should be a good candidate for the generation of vascular or tumor targeting agents in cancer therapy. The cells successfully achieved milligram level production of Nb per 1 L culture (Fig. 1f). Based on *K*_D_ and yield, we choose Nb C184 for subsequent design of CAR. Amino acid sequences of C184 were identified after the VHH genes against CD105 were selected by phage display library (Supplementary Fig. 1).

### Generation of anti-CD105 CAR-T cells

As AAVS1 is a safe genomic location for integrating a gene, we designed guide RNAs (gRNAs) targeting AAVS1 and generated a CRISPR/Cas9 vector. Following transfection into 293T cells, the specific gene knockout was evidenced by PCR and subsequent T7 endonuclease 1 assay (Supplementary Fig. 2a,b).

**Fig. 2:**
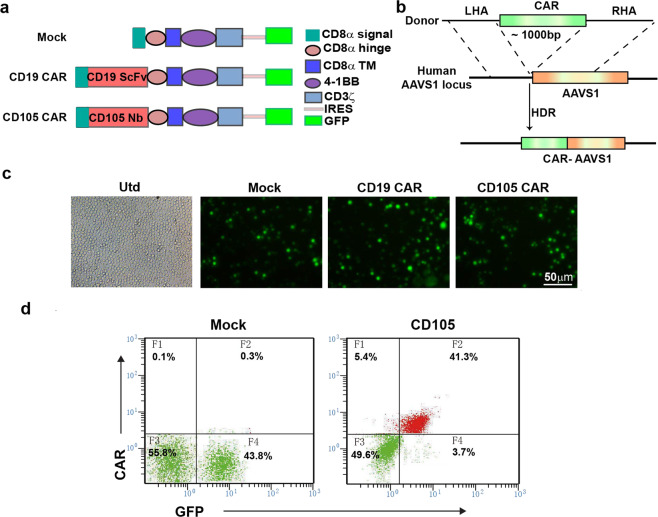
Generation of CD105-chimeric antigen receptor T cells. **a** Schematic illustration of vector expressing CARs, CAR with the signal peptide, anti-CD105 Nanobody (C184), CD8α hige and transmembrane domain, 4-1BB costimulatory endodomain, and CD3ζ signaling domain along with GFP using IRES. **b** HDR-mediated integration of CAR into the AAVS1 locus. Top, donor containing the CAR, CRISPR/Cas9-targeted CAR gene integration into the AAVS1 locus cassette flanked by homology arms. **c** Expression of GFP by fluorescence microscope after electroporation into T cells. **d** CD105-specific CAR expression on human T cells were analyzed using flow cytometry

Simultaneously, the CD105-specific CAR was generated by linking the DNA fragment for the signal peptide, the VHH domain, human CD8a hinge region and transmembrane domains, and 4-1BB and CD3ζ signaling domains with internal ribosome entry site (IRES) and green fluorescent protein (GFP) (Fig. 2a). Following electroporation and homologous recombination, the generated CD105-specific CAR was inserted into AAVS1 site (Fig. 2b).

PBMCs were isolated and after culture for a short period, T cells were enriched and activated for 48 h. The activated T cells were electroporated with pX330-sgRNA targeting the AAVS1 locus and donor-AAVS1 vector. PCR was performed to detect the inserted homologous recombination genes (Mock, *CD19 CAR*, or *CD105 CAR*) Identification of the Mock, CD19 CAR, and CD105 CAR fragments are ~740, 1460, and 1100 bp, respectively (Supplementary Fig. 2c). The results were consistent with the expected target band size, which proved that CAR gene fragment had been successfully integrated into the the AAVS1 locus. Fluorescent microscopy indicated GFP signals in different groups of activated T cells at 24 h post electroporation (Fig. 2c). Flow cytometry was used to detect the expression of the CAR receptor with 41.3% (Fig. 2d) and the ratios of CD4^+^ to CD8^+^ T cells among the different groups of CAR-T cells (Supplementary Fig. 2d).

The levels of CD105 expression in HCC cells (Bel7404, HepG2, SMMC7721, and MHCC97H), human umbilical vein endothelial cell (HUVEC), and 293T cells were characterized by flow cytometry. CD105 was highly expressed on the surface of Bel7404, HepG2, SMMC7721, and HUVEC cells, but little on MHCC97H cells and was not expressed on 293T cells (Supplementary Fig. 3).

**Fig. 3:**
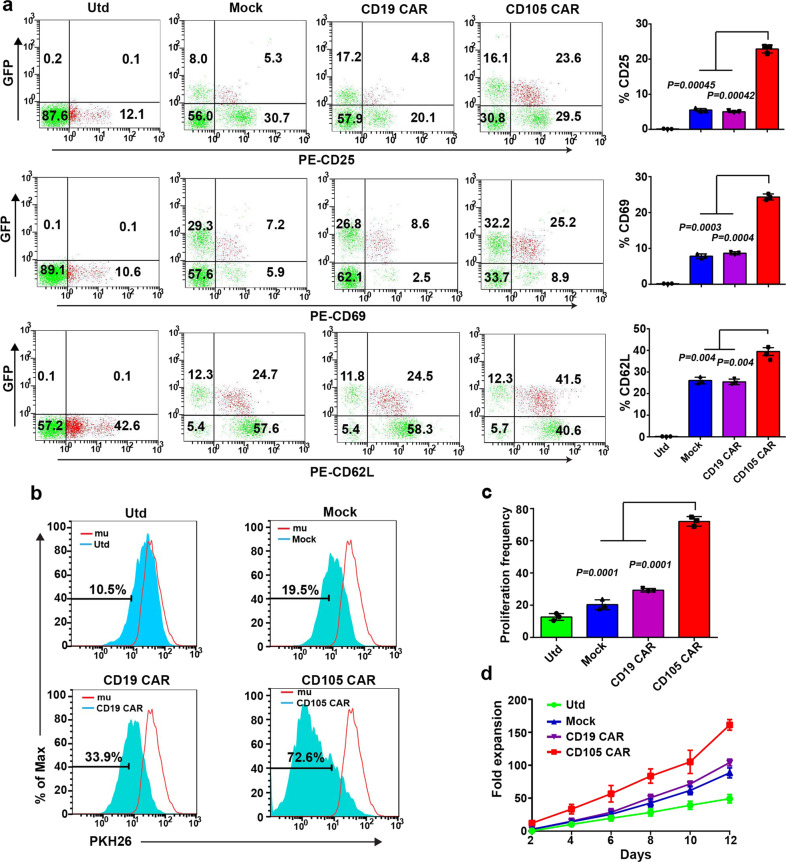
CD105 CAR-T cells undergo activation, antigen-induced differentiation, and proliferation. **a** Anti-CD105 CAR, CD19 CAR, Mock, and the Utd T cells were incubated with CD105^+^ target cells for 3 days; their activation and memory markers were analyzed by flow cytometry. Quantitative analysis of the frequency of activated and memory T cells. **b**, **c** T cells were labeled with PKH26 and incubated with CD105^+^ target cell for 5 days. The proliferation of CD105 CAR-T cells was analyzed by flow cytometry. **d** T cells were labeled with PKH26 and incubated with CD105^+^ cells. The T cells were sampled and counted every other day. Data are present as the mean ± SD from three separate experiments, The quantitative analysis was used by one-way ANOVA with multiple comparisons test

To characterize the anti-CD105 CAR-T cells, anti-CD105 CAR-T cells were challenged with irradiated CD105^+^ target cells (Bel7404) for 72 h. The suspending T cells were collected and stained with fluorescent antibodies. Flow cytometry revealed that the percentages of CD25^+^, CD69^+^, and CD62L^+^ anti-CD105 CAR-T cells were significantly higher than that in the untransfected T (Utd), Mock, and CD19 CAR groups (Fig. 3a). Furthermore, stimulation with CD105^+^ target cells for 120 h significantly promoted the proliferation of PKH26-labeled anti-CD105 CAR-T cells than that of the Utd, Mock, and CD19 CAR groups (Fig. 3b, c). Longitudinal measurements of CD105^+^ target cell-stimulated T-cell proliferation exhibited that the numbers of PKH26-labeled anti-CD105 CAR-T cells were significantly greater than that of other groups in our experimental condition. After 11 days of cultivation, the number of CD105 CAR-T cells increased by 200 times (Fig. 3d). Such data indicated that CD105^+^ target cells further activated anti-CD105 CAR-T cells and promoted their proliferation in vitro.

### CD105^+^ cells stimulate anti-CD105 CAR-T cells activation and pro-inflammatory cytokine production

**Fig. 4:**
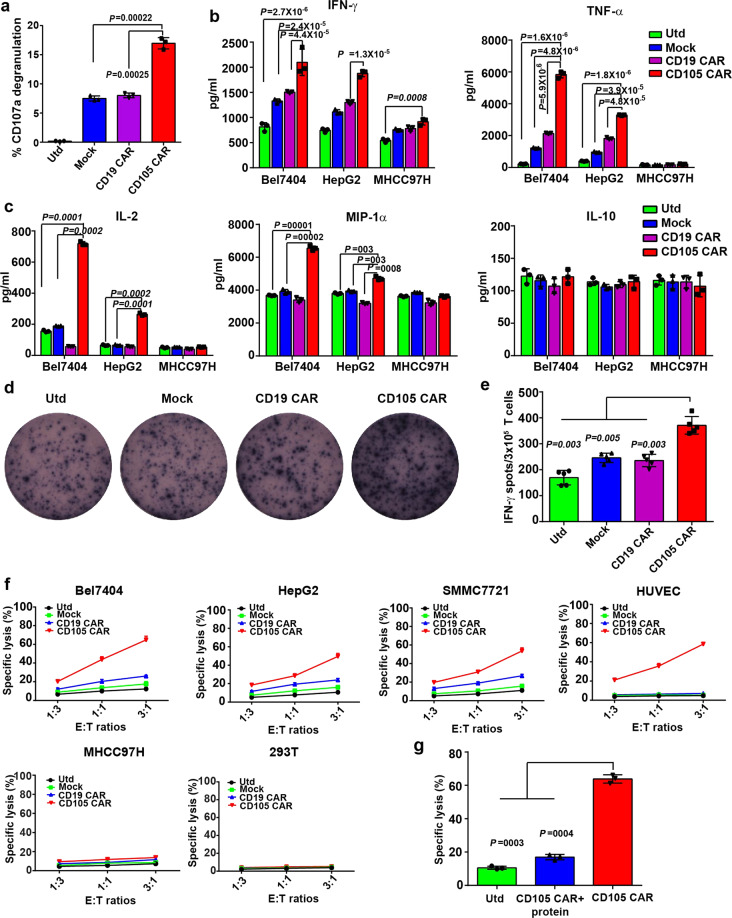
Anti-CD105 CAR-T cells express CD107a, secrete positive regulatory cytokines, and efficiently kill CD105-positive cells. **a** Anti-CD105 CAR-T cells undergo specific degranulation to CD105^+^ cell. The anti-CD105 CAR, CD19 CAR, Mock, and Utd T cells were incubated with tumor cell for 12 h and CD107a degranulation was measured by flow cytometry. The percentages of CD107a^+^ T cells of total anti-CD105 CAR-T cells were significantly higher than that of any other groups. **b**, **c** Anti-CD105 CAR-T cells produced higher levels of cytokines. CAR-T cells and Utd T cells were incubated with tumor cells for 12 h; the levels of cytokines in the culture supernatants were determined by ELISA assay. **d**, **e** ELISPOT analysis of the frequency of IFN-γ-secreted anti-CD105 CAR-T cells. **f** The anti-CD105 CAR-T cells were co-incubated with the indicated cells labeled with PKH26 at E/T ratio 1 : 3, 1 : 1, and 3 : 1 for 16 h and the ratios of PHK26^+^PI^+^ were measured by flow cytometry. Anti-CD105 CAR-T cells had potent cytotoxicity against CD105^+^ target cell and no cytotoxicity against CD105^−^ target cell. **g** CD105 CAR-T was incubated with CD105 protein for 30 min and then co-cultured with Bel7404 at an E/T ratio of 3 : 1 by flow cytometry assay. The results show that the killing effect of CD105 CAR-T on target cells is reduced after incubated with CD105 protein, indicating that CD105 CAR-T cells are targeted to CD105-positive cells. Data show the mean ± SD from three independent experiments. The quantitative analysis was used by one-way ANOVA with multiple comparisons test

We tested whether CD105^+^ target cells could stimulate T-cell activation and pro-inflammatory cytokine production by CD105 CAR-T cells in vitro. The different groups of CAR-T cells were stimulated with the same numbers of Bel7404 cells for 12 h. Flow cytometry analysis indicated that the percentages of CD107a^+^ T cells in the anti-CD105 CAR-T cells were significantly higher than that of other groups (Fig. 4a), which may contribute to their potent cytotoxicity against CD105^+^ cells.

The different groups of CAR-T cells were stimulated with the same number of irradiated Bel7404, HepG2, and MHCC97H for 16 h. The levels of interferon-γ (IFN-γ), tumor necrosis factor-α (TNF-α), interleukin (IL)-2, macrophage inflammatory protein-1α (MIP-1α), and IL-10 in the supernatants of individual groups of cells were measured by enzyme-linked immunosorbent assay (ELISA) (Fig. 4b, c). The levels of IFN-γ, TNF-α, MIP-1α, and IL-2 in the supernatants of cultured anti-CD105 CAR-T cells were significantly higher than that of the Utd, Mock, and CD19 CAR groups of cells following challenged with Bel7404 and HepG2 cells expressed high levels of CD105. There was no significant difference of levels of above cytokines, except for IFN-γ produced by anti-CD105 CAR-T cells cultured with CD105-low expressing MHCC97H cells. However, there was no significant difference in levels of IL-10 among the different groups of cells. Enzyme-linked immune absorbent spot (ELISPOT) revealed that the numbers of IFN-γ-secreting spot-forming cells in the anti-CD105 CAR-T cells were significantly greater than that of the Utd, Mock, and CD19 CAR groups after challenged with CD105^+^ cells (Fig. 4d, e). These independent lines of data demonstrated that anti-CD105 CAR-T cells have activated T cells and secreted higher levels of pro-inflammatory cytokines after challenged with CD105^+^ cells.

### Anti-CD105 CAR-T cells specifically kill CD105^+^ cells in vitro

To test whether anti-CD105 CAR-T cells could specifically kill CD105^+^ target cells, the Utd, Mock, anti-CD19 CAR-T cells, and anti-CD105 CAR-T cells were challenged with PKH26-labeled HCC cells (Bel7404, SMMC7721, HepG2, or MHCC97H), HUVEC, and 293T cells at E/T ratios of 1 : 3, 1 : 1, and 3 : 1 for 16 h. The PKH26-labeled cells were stained with propidium iodide (PI) and the percentages of PKH26^+^PI^+^ dead cells were analyzed by flow cytometry. We found that the anti-CD105 CAR-T cells had potent cytotoxicity against HCC cells (Bel7404, HepG2, and SMMC7721) that expressed high levels of CD105 and CD105-positive HUVEC cells, but little cytotoxicity against CD105-low expressing MHCC97H cells, whereas the Utd, Mock, and anti-CD19 CAR-T cells had low or little cytotoxicity against such HCC cells. All groups showed no cytotoxicity against CD105-negative 293T cells (Fig. 4f). The specific cytotoxicity of anti-CD105 CAR-T cells against CD105^+^ HCC cells was mitigated by pretreatment with with CD105 protein; the results show that the killing effect of CD105 CAR-T on target cells is reduced after incubated with CD105 protein (Fig. 4g). Hence, the anti-CD105 CAR-T cells effectively and specifically killed CD105^+^ cells in vitro.

### Anti-CD105 CAR-T cells inhibit the tumor growth of human tumor xenograft model and prolong the survival of tumor-bearing mice

**Fig. 5:**
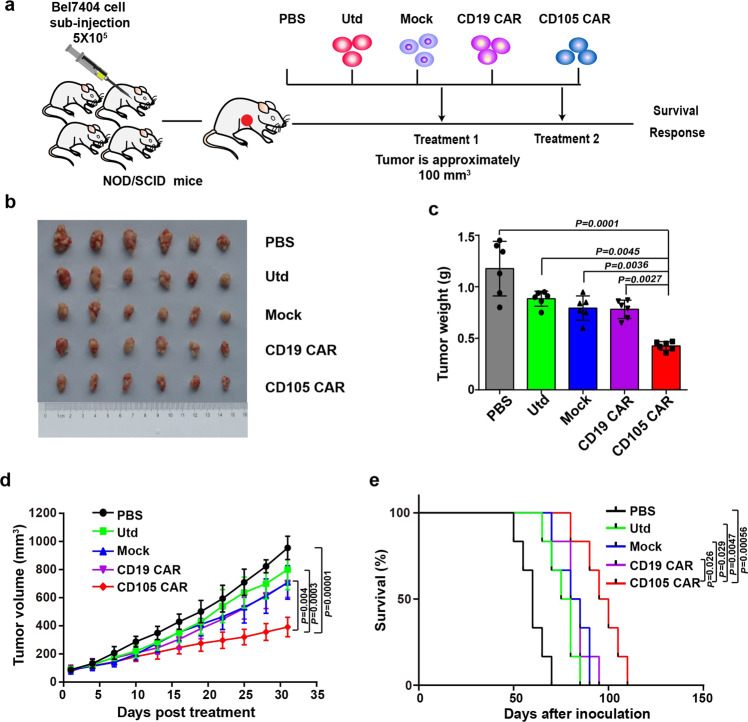
In vivo antitumor activities of anti-CD105 CAR-T cells in the established subcutaneous human tumor xenografts. **a** Experimental schema: NOD/SCID mice were injected subcutaneously with Bel7404 cells (5 × 10^5^ cells/mouse). When the tumor volume reached about 100 mm^3^, the mice were randomized and treated with vehicle alone or with 2 × 10^6^ Utd, Mock, anti-CD19 CAR-T cells, and anti-CD105 CAR-T cells in 0.2 ml PBS (*n* = 6). **b** Bel7404 tumors from the mice killed at the endpoint. **c** The tumor weights were quantified. Anti-CD105 CAR-T cells significantly reduced the tumor weights. **d** The tumor growth curves. Anti-CD105 CAR-T cells significantly reduced the volumes of tumors. **e** The survival of tumor-bearing mice. Anti-CD105 CAR-T cells prolonged the survival of tumor-bearing mice. Data are representative images or expressed as the mean ± SD of each group

Human tumor xenograft models were well-established in NOD/SCID mice by subcutaneous injection with 5 × 10^5^ Bel7404 (Fig. 5a). When the tumor volumes were ~100 mm^3^, the mice were randomized and treated with phosphate-buffered saline (PBS), 2 × 10^6^ Utd, Mock CAR-T (Mock), anti-CD19 CAR-T, and anti-CD105 CAR-T twice. The dynamic growth of individual groups of tumors was monitored longitudinally (Fig. 5b). Treatment with anti-CD105 CAR-T cells significantly reduced the volume and weight of tumor in mice, and prolonged the survival of tumor-bearing mice compared with PBS, Utd, Mock, or anti-CD19 CAR-T cells control (Fig. 5c–e). These data indicated that the intensified in vivo antitumor activity of anti-CD105 CAR-T cells is dependent on the binding of CD105 Nbs to CD105 antigen expressed by tumor cell and tumor vascular endothelial cell in the tumor microenvironment.

**Fig. 6:**
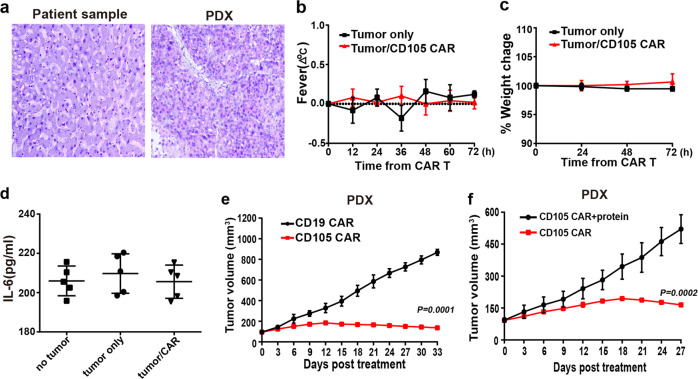
CD105 CAR-T cells efficiently inhibit growth of patient-derived xenograft (PDX) of hepatocellular carcinoma. **a** H&E staining tissue of primary tumor and PDX. The xenograft demonstrates morphology consistent with the original human tumor. The change of body temperature and weight (**b**, **c** *n* = 5) was calculated. The serum levels of IL-6 (**d**) was measured by ELISA (*n* = 5). **e** Growth curve of PDX (*n* = 5) treated with the CD19 or CD105 CAR-T cells at indicated time point. At the end of the experiment, the tumors treated with CD105 CAR-T cells were significantly smaller than CD19 CAR-T group. **f** CD105 CAR-T was incubated with CD105 protein for 30 min, tumor-bearing mice were injected with CD105 CAR -T cells in PDX models. The results show that the antitumor effect of CD105 CAR-T is reduced after incubated with CD105 protein

The histologies of the original tumor tissue (from the patients) and the xenograft tumor tissue (from the tumor transplanted mice) were compared to validate the establishment of the patient-derived xenograft (PDX) model. Our observation on hematoxylin and eosin staining sections found almost none of histological difference between the two different sources of tumor tissue, indicating the similarities of histological characteristics (Fig. 6a). After receiving twice of 2 × 10^6^ CAR-T therapies intravenously, for therapeutic safety evaluation purpose, according to our continuous observation, the mice ate and drank normally. The body temperature of each individual from the different groups was normal, there were no symptoms of fever, and the body weight of the mice did not change significantly (Fig. 6b, c). The serum cytokine IL-6 did not increase significantly with CD105 CAR-T treatment (Fig. 6d). No visible bleeding was observed in organ by autopsy of the PDX mice. The results showed that the mice treated with CD105 CAR-T had no obvious toxic side effects. PDX mice from CD105 CAR-T treatment group showed more significant suppression of tumor growth comparing CD19 CAR-T control group (Fig. 6e). The efficient antitumor effect was observed in the xenograft. CD105 CAR-T was incubated with CD105 protein for 30 min and then were injected into the tumor-bearing mice through the tail vein. The results show that the antitumor effect of CD105 CAR-T is reduced after being incubated with CD105 protein (Fig. 6f). The results showed that CD105 CAR-T could target to therapy the tumor.

**Fig. 7:**
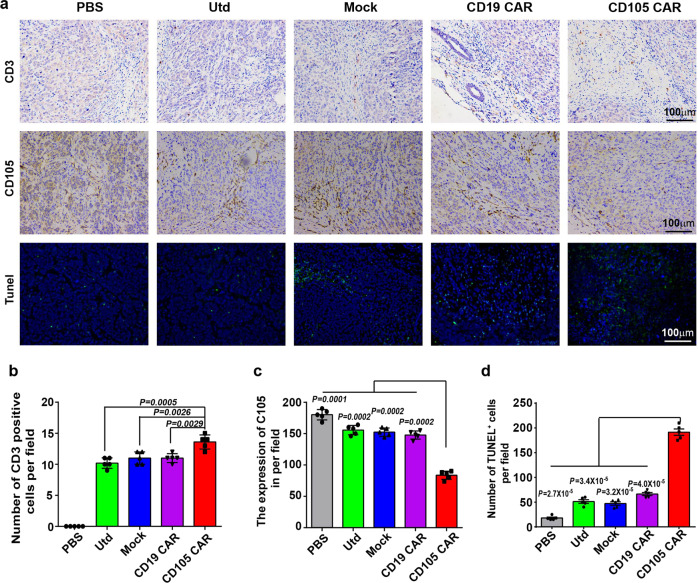
Treatment with anti-CD105 CAR-T cells increased T-cell tumor infiltration, reduced the CD105 expression in the tumor tissues, and increased tumor cells apoptosis. **a** Tumors were collected from mice bearing Bel7404 subcutaneous xenografts treated with CD105 CAR, CD19 CAR, Mock, Utd, or PBS. Formalin-fixed, paraffin-embedded tumor sections were consecutively cut and stained for human CD3 expression, CD105 expression, and TUNEL assays. **b** Quantitative analysis of T-cell infiltrates in tumor tissues. **c** Quantitative analysis of the CD105 expression. **d** Quantitative analysis of apoptotic cells. Data are representative images (magnification ×100) or expressed as the mean ± SD from five randomly selected fields of tumor thin sections. The quantitative analysis was used by one-way ANOVA with multiple comparisons test

Immunohistochemistry exhibited that the numbers of anti-CD3 stained T cells in the tumors from the mice receiving anti-CD105 CAR-T cells were significantly higher than those in other groups and the effects of anti-CD105 CAR-T cells migrating into the tumors tended to be dose dependent (Fig. 7a, b). In comparison with that of other groups, treatment with anti-CD105 CAR-T cells significantly decreased the expression of CD105 in the tumor tissue (Fig. 7a, c). Terminal deoxynucleotidyl transferase dUTP nick end labeling (TUNEL) assays displayed that the numbers of apoptotic cells in the tumors from the mice receiving anti-CD105 CAR-T cells were significantly greater than that of the Utd, Mock, and CD19 CAR groups (Fig. 7a, d). Immunohistochemistry analysis revealed that the numbers of anti-Ki67-stained cells in the mice receiving anti-CD105 CAR-T cells were significantly less than other groups (Supplementary Fig. 4a, b). Anti-CD105 CAR-T cells increased the numbers of anti-Bax-stained apoptotic cells in the tumors from the mice (Supplementary Fig. 4a, c). Collectively, these data indicated that treatment with anti-CD105 CAR-T cells preferably inhibited the growth of implanted human tumors in mice by inducing apoptosis.

We compared lentivirus encoded CD105 CAR-T cells (LV-CD105 CAR-T) with T cells inserted with CD105 CAR at AAVS1 locus (AAVS1-CD105 CAR-T). We first detected the expression of exhaustion markers in CAR-T cells culturing with the presence of antigen. The expression of exhaustion markers in LV-CD105 CAR-T cells was higher than that in AAVS1-CD105 CAR-T cells, indicating that LV-CD105 CAR-T cells had shown signs of depletion (Supplementary Fig. 5a). LV-CD105 CAR and AAVS1-CD105 CAR-T cells differed markedly in their antitumor activity; AAVS1-CD105 CAR-T cells inhibited tumor growth and induced greater responses (Supplementary Fig. 5b). Immunohistochemistry exhibited that the numbers of human anti-CD3-stained T cells in the tumors from the mice receiving AAVS1-CD105 CAR-T cells were significantly higher than those in LV-CD105 CAR-T cells and Utd groups. The result revealed that human CD3^+^ T cells had accumulated in residual tumors after intravenous AAVS1-CD105 CAR-T administration, but less accumulation in LV-CD105 CAR (Supplementary Fig. 5c)

## Discussion

CAR-T cells directly recognize tumor surface antigen and exert their antitumor effects in an major histocompatibility complex (MHC)-independent manner, offering significant advantages compared to conventional anti-cancer immunotherapies.^11,37^ Although CAR-T cell immunotherapies have been successful in various clinical treatments of hematopoietic malignancies, there is still an enormous barrier to the treatment of solid tumors.^15,38^ The major drawbacks are the lack of available specific molecular markers, as well as the difficulty to efficiently reach the local tumor.^39^ To address the barriers of applying CAR-T cells to solid tumor treatment, the discovery of markers and the establishment of a novel type of CAR-T cells with the ability to penetrate and enrich the tumor are urgently needed. CD105 is overexpressed on tumor-associated vascular endothelium cell and tumor, which promotes tumor growth. However, CD105 has not been reported as a target for CAR-T cells in vitro or in vivo.

Given that Nbs have unique structural and molecular characteristics that allows them to easily penetrate into the tumor tissues,^40^ employing Nbs to construct the CAR for CAR-T cells could be an effective strategy. Our study has successfully screened six human CD105-specific Nbs with improved affinity for CD105^+^ target cells. These Nbs displayed quite high affinity and production per liter culture medium that support the demands of further research. Then, we generated the CAR-T cell selectively engineered by one of the six anti-CD105 Nbs, which presented the most favorable expression and affinity in the present study. In the future, this CD105 Nb will be applied as a biomarker for both the diagnosis and the later treatment of tumors. This is the first report about the generation of CAR-T cells based on human CD105-specific Nb targeting CD105^+^ solid tumor.

Compared with retroviral vector transfection system, gene-editing techniques can ensure the accuracy of site-specific insertion to reduce the risk of insertional mutations.^41,42^ CRISPR/Cas9 system allows the precise targeting of specific gene fragments and has been widely used in the field of biomedicine. Some studies have showed successful integration of sequence of the desired genes into certain sites (e.g., TRAC and TCR) of the T-cell genome to construct the CARs by CRISPR/Cas9 system.^34,35^ AAVS1 on human chromosome 19 is a “safe harbor” site and can ensure the transfer of DNA fragments and their functions by CRISPR/Cas9. Previous studies generated transgenic pluripotent stem cells (iPSCs) or immune cells to treat diseases using CRISPR/Cas9-based gene-editing approach by modifying AAVS1 site.^43,44^ In this study, for the first time, we used CRISPR/Cas9 technology to successfully insert the CAR fragment into the AAVS1 site on the genome of T cells. Compared with traditional CAR-T technology, CRISPR/Cas9-editing technology can achieve the site-specific integration of CAR elements in the genome in one step without the use of viruses. This means that the preparation process, production process, and preparation time of CAR-T cells will be greatly simplified and shortened, thus greatly reducing the production cost of CAR-T cells, improving the homogeneity of CAR-T products. Based on the results, we believe that the antitumor effect of AAVS1-CD105 CAR-T cells is better than that of LV-CD105 CAR-T cells in this study.

From the results of flow cytometric analyses, we found that CD105 target cells effectively activated anti-CD105 CAR-T cells in vitro. This was evidenced by the upregulation of CD25 and CD69 expression, evaluated by measuring cell surface CD107a as an early measure of effector activity. Incubation of anti-CD105 CAR-T cells with CD105^+^ target cells resulted in significant degranulation and showed robust activity. The levels of pro-inflammatory cytokines viz IFN-γ, TNF-α, IL-2, and MIP-1α in the culture medium were all increased following co-culture with CD105^+^ target cells, and these anti-CD105 CAR-T cells also displayed significantly higher proliferation, compared to the control group. Accordingly, our study showed that CD105 CAR-T cells effectively killed CD105^+^ target cells but not CD105^−^ cells in vitro. CD105 is rarely expressed on normal blood vessels, except for the umbilical cord of newborns. Cell mortality in vitro showed that CD105 CAR-T cells had no cytotoxicity against CD105-negative cells, so CD105 CAR-T cells are less toxic to normal cells. These data suggested that the CAR-T cells became activated and cytotoxic, and acquired the ability to specifically kill CD105^+^ tumor cells. It is widely accepted that an inflammatory local environment would overcome immune suppression.^29^ Here we found our CAR-T cells produced the elevated cytokine levels that created the necessary environment, in which the CAR-T cells were able to enhance their abilities to conquer the immunosuppression of tumor microenvironment and to lyse the tumor cells.

The in vivo treatment of anti-CD105 CAR-T cells displayed inhibition of tumor growth and prolonged survival periods of human tumor xenograft mice. Immunohistochemitry showed a decreased number of cells with the proliferative marker Ki67 and the expression of CD105, an increased tumor cell apoptosis, and the expression of apoptosis protein Bax. The CD105-specific Nb-based CAR-T cells were found to be activated and infiltrated in the tumor of xenograft mice, confirmed by the higher levels of anti-human CD3-positive staining cells in tumor tissues following the anti-CD105 CAR-T cell treatment. The infiltration of anti-CD105 CAR-T cells may play a disruptive role in tumor angiogenesis and the hampering of tumor cell proliferation in the local region.

The favorable antitumor efficacy of anti-CD105 CAR-T cells in the human tumor xenograft models in our study, which was not reported before. Nb-based CAR-T cells that target aspects of the tumor might counteract multiple cancer types and model. Xenograft models on immunodeficient mice (e.g., NOD/SCID mice) are commonly used for functional research of CAR-T cells. Xenografts that create exogenous human tumor in the mice in our study, although have some drawbacks, would still provide more information with similar histopathological and immunological features to the actual human primary tumor in certain range rather than allografts and might lay the base for further trials.^45^

The PDX model has several advantages over the cancer cell-derived xenotransplantation murine model. Previous studies have shown that the tumors in the PDX model are biologically stable and can accurately reflect the histopathology, gene expression, and treatment response. Thus, PDX models may provide a method for precise preclinical evaluation for novel CAR-T immunotherapies.^46^ There was a report that GPC3-positive PDX mouse model for HCC was well-established and characterized, and showed significantly suppressed tumor growth by treatment of GPC3-CAR-T cells.^47^ Therefore, we further performed CAT-T treatment by using the PDX model in vivo to test the efficacies and safety of CD105 CAR-T cell therapy. In this study, CD105 CAR-T played efficient therapeutic effect in the PDX model, this suggest that CD105 CAR-T cells may be promising for intervention of CD105^+^ HCC and other malignancies. Cytokine release syndrome (CRS) is a toxic side effect of CAR-T therapy; CRS is characterized by fever and hypotension associated with elevated serum cytokines (such as IL-6).^48^ The results showed the serum cytokine IL-6 did not increase significantly with CD105 CAR-T treatment and no symptoms of fever was observed, indicating that CD105 CAR-T treatment showed no obvious toxicity in mice. The efficacy of CD105 CAR-T in the treatment of solid tumors still requires further optimization and explority use on other tumor types as an extension of this work before clinical trials, such as injection methodology and the dosage (CAR-T cell number) of administration, which required validatation by using some additional models. CD105 CAR-T cells designed to secrete immunosuppressive agents or cytokines are also worthy of further exploring their therapeutic effects on tumors.

Altogether, we successfully generated a novel CD105-specific Nb and, for the first time, established human CAR-T cells by inserting this Nb as the CAR fragment into the safe site of the genome in human T cells using CRISPR/Cas9 technology. The generated anti-CD105 CAR-T cells displayed potent cytotoxicity towards CD105^+^ tumor cell growth in vitro, and significant inhibition against solid tumors via specifically targeting human CD105 on tumor and tumor vascular endothelial cell of the microenvironment in the xenograft mice models. This preclinical study presents not only a new strategy but also a promising prospect to generate effective CAR-T cells for anti-solid tumor cancers. Incorporation with other types of therapeutic approaches, such as checkpoint inhibitors or with cytokine-secretable CAR-T cells based on other new targeting Nbs, may help improve the preclinical outcomes of solid tumor.

## Materials and methods

### Cell culture

Human HCC cells HepG2, HUVEC, and non-tumor embryonic kidney (293T) cells were obtained from the American Type Culture Collection. HCC cell lines Bel7404, SMMC7721, and MHCC97H cell were obtained from the Cell Bank of Chinese Academy of Sciences (Shanghai, China). The cells were cultured in Dulbecco’s modified Eagle’s medium medium supplemented with 10% fetal bovine serum (FBS), 100 U/ml penicillin, and 100 μg/ml streptomycin (complete medium, Invitrogen) at 37 °C in 5% CO_2_.

### Construction and binding analysis of CD105 Nb

A healthy young camel was injected subcutaneously with extracellular domain of CD105 protein (1 mg in PBS) mixed with an equal volume of Freund’ s complete adjuvant on 7-day interval for 6 weeks. Six weeks later, PBMCs were collected, the total RNA was extracted, and cDNA was synthetized with a RevertAid First Strand cDNA Synthesis Kit. The VHHs were amplified by nested PCR.^49^ The pMECS phagemid vector and the nested PCR product were digested with NotI and PstI restriction enzymes, and then ligated with T4 DNA ligase enzyme at 16 °C overnight. The ligated vector was electroporated into electrocompetent *Escherichia coli* TG1 cells. The electroporated cells were cultured. The VHH libraries were displayed on phages after infection with VCSM13 helper phages. The Nbs against CD105 were screened by phage display; after three rounds of screening, the positive clones were identified by the periplasmic extract ELISA, the plasmids from positive colonies were extracted, and sent for sequencing.^50^ The positive clones were classified into different families, based on the diversity of their amino acid sequences in the CDR3 region. The recombinant pMECS plasmids of the different Nb families were extracted from TG1 cells and transformed into *E. coli* WK6 cells by electroporation. The expression of Nbs was induced by isopropyl *β*-d-1-thiogalactopyranoside. The specific Nbs containing His-tags were purified from the cell lysate by immobilized metal affinity chromatography using a His-select column and analyzed by 15% SDS-PAGE.

The affinities of selected anti-CD105 Nbs were measured by the kinetic analysis by surface plasmon resonance experiment with PlexArray® HT system.^51^ First, CD105 Nbs (10 μg mL^−1^) were immobilized on PlexArray® Nanocapture® Sensor Chips. Then, to take note of the signals, different concentrations were injected with a flow rate of 3.0 μL s^−1^. Finally, a regeneration step was performed after the affinity binding reaction. Briefly, the sensor chips were regenerated with 0.05 mol L^−1^ NaOH for 2 min and then PBS buffer was injected over the sensor surface for about 5 min. All signals were recorded as sensorgrams and steps were performed in triplicate at 25 °C.

### Construction of CRISPR/Cas9 vectors and HDR vectors contained CAR sequence

Three 20 bp gRNA (Supplementary Table 1) were designed for the *PPP1R12C* gene at the AAVS1 locus on chromosome 19. These gRNA oligonucleotides were synthesized by Shanghai Biotech (Shanghai, China). After annealing the gRNA, it was ligated to the plasmid pX330. After being sequenced, the generated pX330-sgRNAs were transfected into 293T cells and their genomic DNA was extracted for PCR amplification of the DNA fragment covering the knockout site. The specific gene-editing efficiency was analyzed by T7 endonuclease 1 assay.

DNA sequence of the desired CAR was cloned into KpnI and HindIII sites of the plasmid Puc-Mdonor-AAVS1 (Genomeditech, Shangai, China) containing the homologous recombinant arms to construct homology-directed repair (HDR) vectors. As shown in Fig. 2a, the CD105 CAR was composed of the CD105 Nb, human CD8a hinge, and transmembrane domain (nucleotides 412–609), the intracellular signaling domain of CD137 (4-1BB, nucleotides 640–765), and CD3ζ molecules (nucleotides 154–492). Individual CAR sequences were linked to GFP through IRES.

### Isolation of human T cells and generation of CD105-specific CAR-T cells

Peripheral blood samples were collected from healthy human donors and PBMCs were prepared by Ficolle-Paque density gradient centrifugation. PBMCs were cultured in RPMI-1640 medium (Gibco) containing 10% FBS, 100 U/ml penicillin, and 100 µg/ml streptomycin (complete medium). After 2 h culture, the suspended lymphocytes were collected and cultured in complete RPMI-1640 medium containing 100 U/ml of recombinant human IL-2 (Peprotech, USA). The cells were stimulated for 24 h using the Easy-T Stimulation Kit (Jikai, Shanghai), according to the manufacturer’s instructions. Two days after stimulation, lymphocytes (1 × 10^6^ cells/tube) were transfected with 2 µg pX330-sgRNA and 4 µg individual types of Puc-Mdonor-AAVS1 vector containing Mock, CD19 CAR, or CD105 CAR by electroporation in the Lonza electroporation mixing buffer using a Lonza 4D Necleofector^TM^ electroporation system with preset program. Subsequently, the cells were cultured in pre-warm complete RPMI-1640 medium for 4 h and continually cultured in the medium containing 500 U/ml of IL-2 for 48 h. The fluorescent signals were observed under a fluorescent microscope (Nikon, Japan).

For identification of CAR genes in T cell, total DNA was extracted from anti-CD105 CAR-T (CD105 CAR), anti-CD19 CAR-T (CD19 CAR), or Mock-T cells, and the CAR genes inserted into the AAVS1 locus were identified by PCR using specific primers. CAR fragment primers with the sequences were CMV-F: 5′-CGCAAATGGGCGGTAGGCGTG-3′ (located upstream of the CAR gene insertion site) and AAVS1-R: 5′-ATGGGGCTTTTCTGTCACCA-3′ (located downstream of the CAR gene insertion site). The PCR reactions were performed in duplicate at 94 °C for 2 min and subjected to 35 cycles of 94 °C for 30 s, 58 °C for 30 s, and 72 °C for 30 s, followed by a final extension at 72°C for 2 min. The PCR products were visualized by 1% agarose gel electrophoresis.

Lentivirus encoded CD105 CAR-T cells (LV-CD105 CAR-T) were prepared as follows: recombinant lentiviral particles were produced by a calcium phosphate transfection system. Lentiviral particles were concentrated 30-fold by followed by ultracentrifugation at 28,000 r.p.m. 16 °C for 120 min. Activated T cells were then transduced with the lentiviral vector at a multiplicity of infection of 10 U/cell. The transduced T cells were cultured at a concentration of 5 × 10^5^ cells/mL in the presence of recombinant human IL-2 (100 U/mL) every other day.

### Flow cytometry assay

Four days after electroporation, T cells incubated with Phycoerythrin (PE)-conjugated anti-His-tag antibody, flow cytometry was used to detect the expression of the CAR receptor. Furthermore, individual groups of CAR-T cells and control Utd T cells were stained with PE-anti-CD25 (BD, Cat555431), PE-anti-CD69 (BD, Cat555531), or PE-anti-CD62L antibody (BD, Cat555543). Some cells from each group were stained with PE-anti-CD4 (BD, Cat555347) and percp-cy5.5-anti-CD8 antibody (BD, Cat565310). The percentages of CD4^+^ and CD8^+^ T cells in individual groups of CAR-T cells were determined by flow cytometry. In addition, human HCC cells (Bel7404, HepG2, SMMC7721, and MHCC97H), HUVEC, and 293T cells were stained with PE-anti-CD105 antibody (eBioscience, Cat12-1057-42). The percentages of CD105^+^ target cells was examined by flow cytometry. The data were analyzed using FlowJo software (7.6).

### Cell proliferation and cytotoxicity assay in vitro

Proliferation of T cells was determined by PKH26 staining in vitro. Individual groups of CAR-T and control Utd T cells (1 × 10^6^ cells/tube) were labeled with PKH26 (Sigma-Aldrich) at 37 °C for 5 min. After being washed, individual groups of cells were stimulated with the same number of CD105^+^ target cells that had been irradiated (100 Gy) for 120 h. The suspended T cells were collected and the percentages of proliferative T cells were determined by flow cytometry.

For cytotoxicity assays, individual groups of CAR-T and control Utd T cells were cultured in triplicate with each type of PKH26-labeled target cells at a ratio of 3 : 1, 1 : 1, or 1 : 3 for 16 h. After being washed, the adhered target cells were collected and stained with PI stain (Sigma), and the percentages of PKH26^+^PI^+^ dead cells in individual groups were determined by flow cytometry as % of specific lysis. In addition, anti-CD105 CAR-T cells were co-cultured with Bel7404 cells at a ratio of 3 : 1 for 16 h in the presence or absence of CD105 protein (eBioscience, Cat14-1051-85). The percentages of lysed target cells were determined by flow cytometry. Moreover, individual groups of CAR-T and control Utd T cells were cultured in triplicate with Bel7404 cells at a ratio of 3 : 1 for 12 h and stained with PE-anti-CD107a antibody (Biolegend,Cat304124). The percentages of CD107a^+^ T cells were determined by flow cytometry.

### ELISA and ELISPOT assays

Individual groups of CAR-T and control Utd T cells (1 × 10^5^ cells/well) were stimulated with 1 × 10^5^ cells/well of Bel7404, HepG2, or MHCC97H cells for 16 h. The levels of IFN-γ, TNF-α, IL-2, MIP-1α, and IL-10 in the supernatants of cultured cells were determined by ELISA kits (BD), according to the manufacturer’s protocols.

To test the density of IFN-γ-secreting cells, ELISPOT assay was performed. Briefly, individual groups of CAR-T and control Utd cells (3 × 10^5^ cells/well) were stimulated in triplicate with 10^5^ irradiated Bel7404 cells at 37 °C overnight in 96-well plates that had been coated with anti-IFN-γ. After being washed, the captured IFN-γ in individual wells was reacted with biotinylated anti-IFN-γ at 4 °C overnight. Subsequently, the IFN-γ-specific immunocomplex was detected with streptavidin-AP and visualized using a substrate solution 5-Bromo-4-Chloro-3-Indolyl Phosphate (BCIP)/Tetranitroblue tetrazolium chloride (NBT). The numbers of spot-forming cells in individual wells were analyzed on a CTL ImmunoSpot S6 Ultimate-V analyzer using Immunospot software, version 5.1.

### Establishment of human tumor xenograft models

Female NOD/SCID mice were purchased from Vitalriver (Beijing, China) and housed in a specific pathogen-free facility with free access to autoclaved food and water. The mice were randomized and injected subcutaneously with 5 × 10^5^ Bel7404 cells in their right flank to induce a solid tumor. When individual mice developed a tumor with a volume of ~100 mm^3^, the mice were randomized and treated intravenously with PBS, 2 × 10^6^ Utd T, Mock CAR-T, anti-CD19 CAR-T, and anti-CD105 CAR-T twice. The dynamic growth of implanted tumors was monitored every 3 days using a caliper through measurement of the length (*L*) and width (*W*), and the tumor volumes were calculated by the formula of (*L* × *W*^2^)/2. Post treatment, the mice were killed and their tumors were dissected, photo-imaged, and weighed. Another set of mice was continually monitored until death and the survival of individual groups of mice was calculated. Similarly, tumor-bearing mice were treated with 2 × 10^6^ LV-CD105 CAR-T cells injected into the tail vein. The experimental protocols were approved by the Animal Research and Care committee of Guangxi Medical University, Nanning, China.

To generate PDX mouse models of HCC, surgically removed specimens were obtained from HCC patients with informed consent and institutional approval. The human cells and tissue relevant studies were approved by the Ethics Committee of Guangxi Medical University and the First Afliated Hospital of Guangxi Medical University. The collected primary HCC tumor tissues were diced into thin pieces of 3–4 mm and then subcutaneously inoculated into the right flank of 6-week-old male NOD/SCID mice. For continuous transplantation, tumor-bearing animals were anesthetized with ether and killed by cervical dislocation. Tumors were shredded under sterile conditions and serially transplanted into NOD/SCID mice as described above. When tumor volume of individual mice expandedto ~80 ~ 100 mm^3^, the mice were randomized and treated intravenously with 2 × 10^6^ of CD105 CAR-T cells or control CD19 CAR-T cells twice. CD105 CAR-T was incubated with CD105 protein for 30 min and then were injected into the tumor-bearing mice through the tail vein. The size of the tumor was measured every 3 days, and the temperature and body weight of mice were also monitored.

### Immunohistochemistry

The tumor tissues from individual mice were dissected, fixed in 10% neutral formalin, and paraffin-embedded. Furthermore, the tumor tissue sections (4 µm) were stained with anti-Ki67 antibody (kit-0005), anti-CD105 antibody (MAB-0344), anti-Bax antibody (MAB-0254), or anti-CD3 antibody ((kit-0005) from Maixin Biotech. The bound antibodies were reacted with biotinylated second antibodies and detected using the Streptavidin-Peroxidase kit and 3,3′-Diaminobenzidine (Maixin Biotech). In addition, the frequency of apoptotic cells in tumor tissue sections was determined by TUNEL assay using the In Situ Cell Death Detection Kit (fluorescein isothiocyanate (FITC)), Roche, Switzerland), according to the manufacturer’s instruction. Images were obtained under a microscope (Nikon, Japan).

### Statistical analysis

The flow cytometry data was analyzed with FlowJo 7.6 software. Statistical analysis was performed by using GraphPad Prism software 6.0. One-way analysis of variance (ANOVA) with Tukey’s multiple comparison was performed to assess the differences among different groups in the in vitro assays. Tumor growth curve was analyzed using two-way ANOVA with correction for Tukey’s multiple comparison. The survival curve of animals in the difference between groups was calculated by the Kaplan–Meier analysis (log-rank test) log-rank test. A *P*-value < 0.05 was considered statistically significant.

## Supplementary information

Nanobody-based chimeric antigen receptor T cells designed by CRISPRCas9

## Data Availability

The datasets generated during and/or analyzed during the current study are available from the corresponding author upon reasonable request.
