# Supplementary material for: Nanobody-based chimeric antigen receptor T cells designed by CRISPR/Cas9 technology for solid tumor immunotherapy
Source: Signal Transduct Target Ther. 2021 Feb 25;6:80. doi: 10.1038/s41392-021-00462-1 (PMC7904846; doi:10.1038/s41392-021-00462-1)
Supplement: Supplementary file 1 — Nanobody-based chimeric antigen receptor T cells designed by CRISPRCas9 [file 41392_2021_462_MOESM1_ESM.docx]

Supplementary Materials for

**Nanobody-based chimeric antigen receptor T cells designed by CRISPR/Cas9 technology for solid tumor immunotherapy**

Fengzhen Mo ^1, 2*^, Siliang Duan ^1*^, Xiaobing Jiang^3*^, Xiaomei Yang ^1,4^ , Xiaoqiong Hou ^1,4^, Wei Shi ^1^, Cueva Jumbo Juan Carlos ^1^, Aiqun Liu ^1^, Shihua Yin^1^, Wu Wang ^1^, Hua Yao ^1,5^, Zihang Yu^1^, Zhuoran Tang ^1^, Shenxia Xie ^1,2^, Ziqiang Ding^1^, Xinyue Zhao^1^, Bruce D. Hammock ^6#^,Xiaoling Lu ^1,5#^

^1^ International Nanobody Research Center of Guangxi, Guangxi Medical University, Nanning, Guangxi, 530021, China

^2^ Pharmaceutical College, Guangxi Medical University, Nanning, Guangxi, 530021, China

^3^ Department of Neurosurgery, Union Hospital, Tongji Medical College, Huazhong University of Science and Technology, Wuhan, Hubei, 430022, China

^4^ School of Preclinical Medicine, Guangxi Medical University, Nanning, Guangxi, 530021, China

^5^ College of Stomatology, Guangxi Medical University, Nanning, Guangxi, 530021, China

^6^ Department of Entomology and Nematology and UCD Comprehensive Cancer Center, University of California Davis, Davis, CA 95616¬8584，USA

These authors contribute to the equal work.

Correspondence to: [luxiaoling@gxmu.edu.cn](mailto:luxiaoling@gxmu.edu.cn), [bdhammock@ucdavis.edu](mailto:bdhammock@ucdavis.edu)

**This PDF file includes:**

Table. S1

Figures. S1 to S5

**Table S1** **gRNA sequences used in study**

| Name | Forward oligo 5’-3’ | Reverse oligo 5’-3’ |
| --- | --- | --- |
| AAVS1 gRNA1 | CACCGTCCCCTCCACCCCACAGT | AAACACTGTGGGGTGGAGGGGAC |
| AAVS1 gRNA2 | CACCGGGCCACTAGGGACAGGAT | AAACATCCTGTCCCTAGTGGCCC |
| AAVS1 gRNA3 | CACCGACAGAAAAGCCCCATCCTT | AAACAAGGATGGGGCTTTTCTGTC |

**
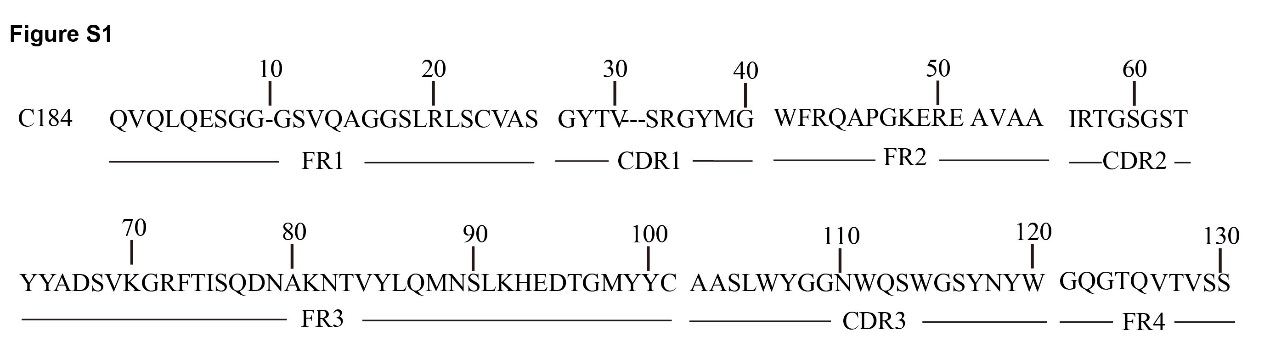
**

**Figure S1.**

Amino acid sequences of anti-CD105 VHHS were identified after the VHH genes against CD105 were selected by phage display library. Amino acid sequence alignment of specific Nb families as classified by CDR3. Amino acids positions of the framework region (FR) and of the three antigen-binding loops (CDR1, CDR2 and CDR3) are numbered according to the IMGT scientific chart for the V-Domain and are indicated at bottom.

**
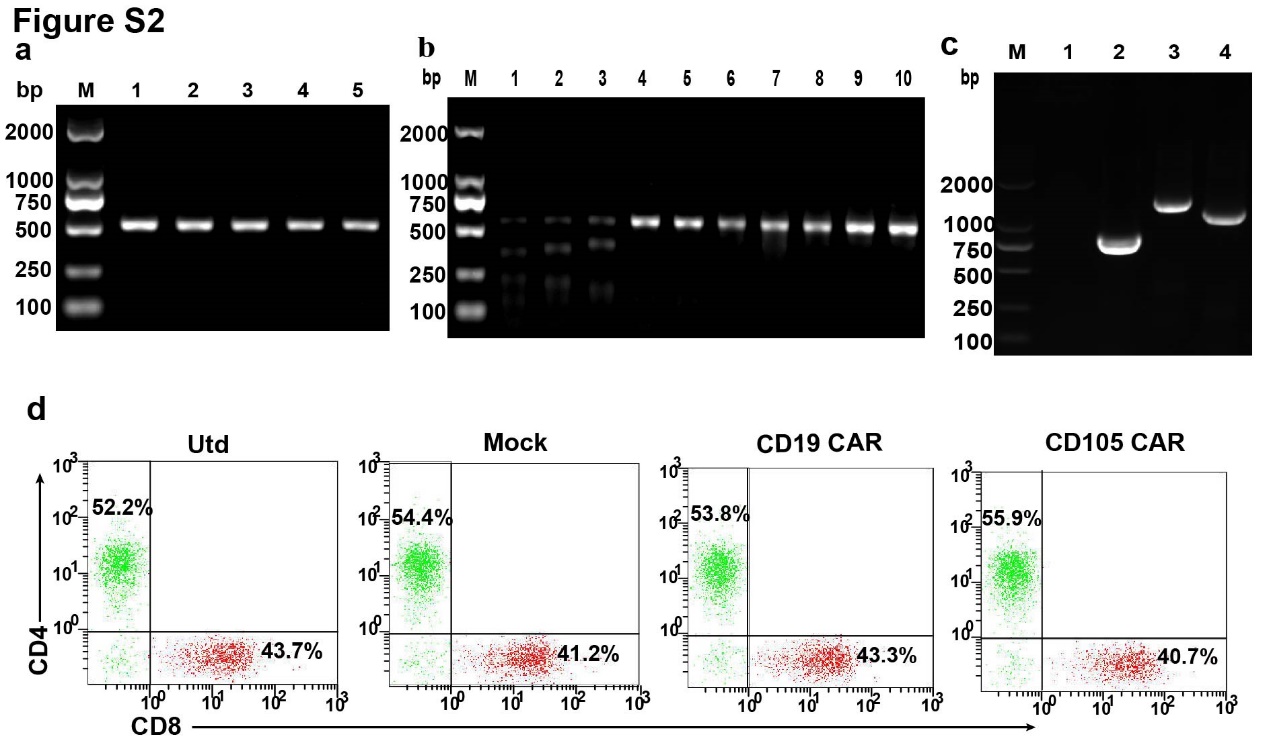
**

**Figure S2.**

**Generation of anti-CD105 CAR-T cells.** (a) PCR-based amplification of fragments containing gRNA targets to produce a fragment size of approximately 500 bp. (b) T7E1 assay indicates that gRNA has a targeted knockout for the AAVS1 locus, Lanes 1-3, the T7E1 digestion generated two anticipated fragments, but not in lanes 4-5 without pX330-sgRNAs vectors transfection, and the lanes 6-10 without T7E1 as negative controls. (c) The inserted homologous recombination genes (Mock, CD19 CAR or CD105 CAR) in T cells after electroporation was detected by PCR. Identification of the Mock ,CD19 CAR, CD105 CAR fragments are approximately 740 bp, 1460 bp and 1100 bp, respectively. M: 2000 bp marker; 1: Utd; 2: Mock; 3: CD19 CAR; 4: CD105 CAR. (d) CD8^+^ and CD4^+^ CAR T cell ratios were detected by flow cytometry. There is no significant difference in the percentages of CD8^+^ and CD4^+^ T cells among CD105 CAR, CD19 CAR, Mock and Utd T cells.

**
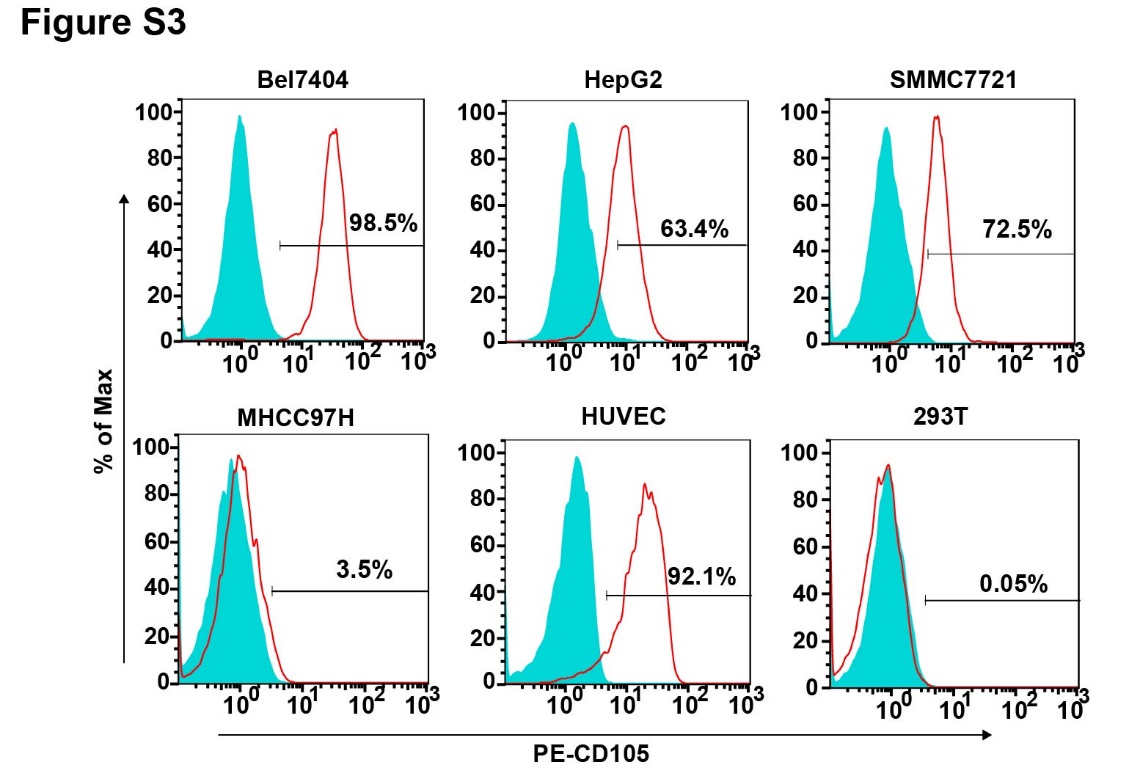
**

**Figure S3.**

**Expression of CD105 on tumor cells and Human umbilical vein endothelial cells**. Representative histograms of surface CD105 expression (red line) on various human HCC cells and normal cells detected by flow cytometry and isotype control (filled blue histograms). CD105 was highly expressed on the surface of Bel7404, HepG2, SMMC7721 and HUVEC cells, but little on MHCC97H cells and no expressed on 293T cells. Data are representative streaming diagram from three separate experiments.


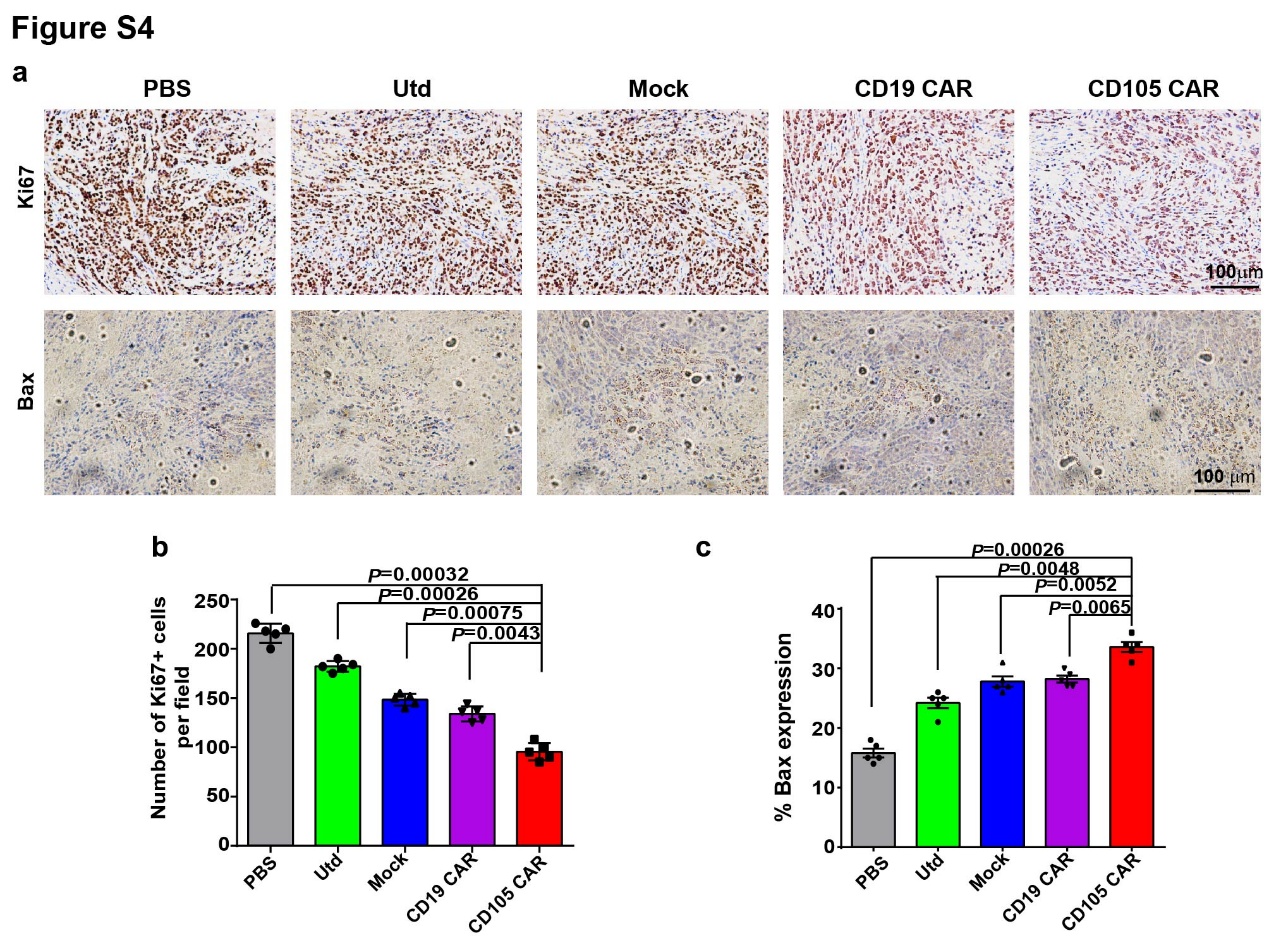


**Figure S4.**

**Treatment with anti-CD105 CAR T cells decreased the proliferation of tumor cells and enhanced the Bax expression in tumor tissues.** (a)Immunohistochemistry analysis of tumor cell proliferation. The different groups of tumor sections were characterized by immunohistochemistry using anti-Ki67 antibodies for tumor cell proliferation and using anti-Bax antibody for Bax expression. (b)Quantitative analysis of Ki67^+^ proliferative tumor cells. (c) Quantitative analysis of the frequency of Bax^+^ cells. Data are representative images (magnification × 100) or expressed as the mean ± SD from 5 randomly selected fields of tumor thin sections. The Quantitative analysis was used by One-Way ANOVA with multiple comparisons test.


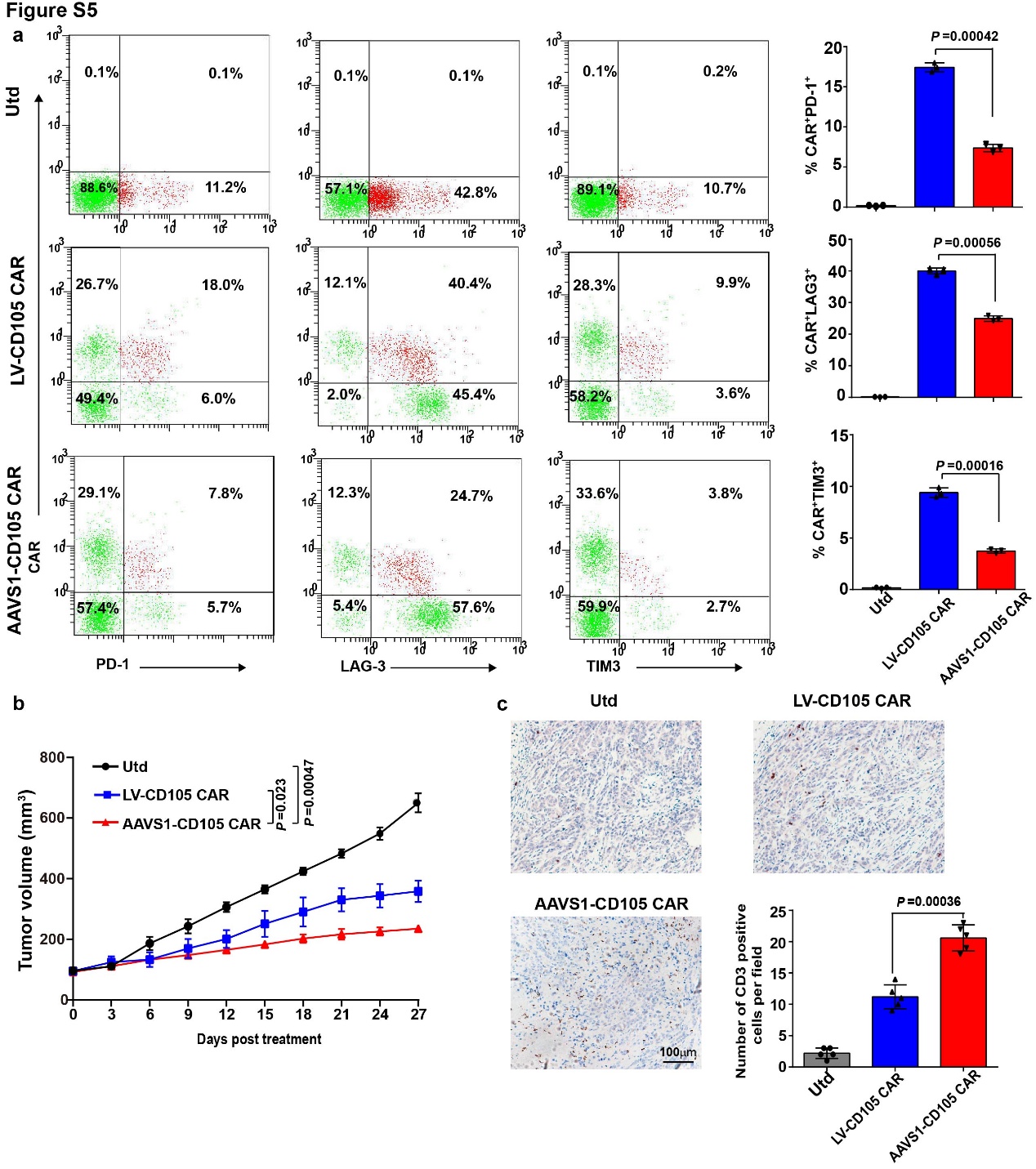


**Figure S5.**

**AAVS1-CD105 CAR T cells outperform conventional CAR T cells by preventing exhaustion in vitro and in vivo.** (a) The expression of exhaustion markers in LV-CD105 CAR T cells was higher than that in AAVS1-CD105 CAR T cells, indicating that LV- CD105 CAR T cells had shown signs of depletion. Data are present as the mean ± SD from three separate experiments. (b)LV-CD105 CAR and AAVS1-CD105 CAR T cells differed markedly in their anti-tumour activity(n=5). (c)Immunohistochemistry exhibited that the numbers of anti-CD3 stained T cells in the tumors from the mice receiving AAVS1-CD105 CAR-T cells were significantly higher than those in LV-CD105 CAR T cells and Utd groups. Data are representative images (magnification × 100) or expressed as the mean ± SD from 5 randomly selected fields of tumor thin sections. The Quantitative analysis was used by One-Way ANOVA with multiple comparisons test.
